# Prevalence of noroviruses in children hospitalized for acute gastroenteritis in Hohhot, China, 2012-2017

**DOI:** 10.1186/s12879-019-4230-x

**Published:** 2019-07-09

**Authors:** Hui-ying Li, Yu-geng Zhang, Xia Lei, Jian Song, Zhao-jun Duan

**Affiliations:** 1 National Institute for Viral Disease Control and Prevention, Chinese Center for Disease Control and Prevention, 155 Changbai Rd., Changping District, Beijing, People’s Republic of China; 2 Center for Disease Control and Prevention of Inner Mongolia Autonomous Region, Inner Mongolia010031, Hohhot, China

**Keywords:** Norovirus, Children, Acute gastroenteritis, Genotype

## Abstract

**Background:**

Noroviruses (NVs) are an important cause of acute gastroenteritis (AGE) worldwide. There are limited data on the prevalence and molecular characterization of NVs in children in Hohhot, China.

**Methods:**

Between January 2012 and December 2017, 1863 stool samples were collected at Maternal and Child Health Hospital in Hohhot. All samples were screened for NVs by real-time reverse transcription polymerase chain reaction (real-time RT-PCR).

**Results:**

NVs were detected in 24.15% of these inpatient cases, ranging from 12.78 to 32.92% in different years. NV was detected throughout the year, with a peak in winter. Based on sequence analysis of the partial *VP1* gene, the 306 identified NV strains were divided into six genotypes: GII.3 (71.24%), GII.4 (23.53%), and GII.2, GII.5, GII.6, and GII.13 (total 5.23%). Based on further sequence analysis of the RNA-dependent RNA polymerase (RdRp), GII.P12/GII.3, GII.Pe/GII.4, and GII.P4/GII.4 were identified as predominant genotypes, accounting for 92.6% of genotyped strains. The median age of the children with NV infection was 8.0 (range 0–59) months. However, children infected with GII.3 were younger (median 7.0 months) than GII.4-positive patients (median 10.0 months).

**Conclusion:**

NV contributed greatly to AGE among hospitalized children in Hohhot in China. Continuous surveillance is important for understanding the local prevalence and characterization of NV.

## Background

NVs are a leading cause of epidemic gastroenteritis [1], responsible for at least 90% of all non-bacterial gastroenteritis cases and 50% of all gastroenteritis outbreaks worldwide [2, 3]. All age groups can be infected by NVs, especially children, the old, and the immunosuppressed. The acute gastroenteritis which caused by NVs has become the second infectious disease due to its highly transmission with fecal-oral route and lowly infectious dose [4, 5]. In the developing countries, more than 200,000 deaths per year are caused by NVs infection [6].

NVs are belonging to *Caliciviridae,* with a 7.5-kb-long ssRNA genome. Three different open reading frames (ORFs) encode different proteins of NVs [7]. Six non-structural proteins are encoded by ORF1, such as p28, VPg, 3CL^pro^, and the structural proteins are encoded by ORF2 (VP1) and ORF3 (VP2) to form the capsid of NVs. [7]. Total seven genogroups (GI, GII, GIII, GIV, GV, GVI, and GVII) NVs are classified by the identities of VP1 and RdRp amino acid sequences of NVs [8, 9]. Human beings are the major host to genogroups GI, GII, and GIV NVs, however, other genogroups NV are mainly infected with animals. The epidemic of human acute gastroenteritis are mainly caused by GI or GII NVs [8, 10] and the recombination between ORF1 and ORF2 [11], antigenic variation of P domain of VP1 [12] are contributed to generate novel NV strains. The emerging of NVs leading to the epidemic of human acute gastroenteritis in the worldwide,at hence, it is important to determine both the capsid and RdRp genotypes for strain characterization.

Despite the large number of NV genotypes co-circulating in children over the past decade, genotypes GII.4 and GII.3 have predominated worldwide [13–17]. Up to nowadays, six outbreaks of acute gastroenteritis are associated with at least seven distinct GII.4 NV variants [14]. Three major clusters (I–III) and five smaller genetic lineages (A–E) have been identified in the VP1 region of GII.3 NV [13, 18]. Global surveillance data over the years indicated that the majority of outbreaks of NV were caused by GII.4, but the recent NV outbreak strains in Asia were GII.2 and GII.17 [1, 19, 20]. Furthermore, other genotypes were co-circulating, with different epidemiological patterns in different regions [1, 17].

China is a vast country, and the prevalence of NV varies by region [1, 19, 21, 22]. However, reports and knowledge of routine sporadic NV disease in northern China have been limited. Furthermore, in several recent years, rare NV genotypes (GII.2, GII.6, and GII.17) have caused prevalent outbreaks in China [19, 20] and other parts of Asia [23, 24]. Therefore, it is important to explore the NV strains in sporadic AGE cases, which may also aid in understanding the prevalence of human NV in the population. In the present study, we explored the epidemiological and molecular characteristics of NVs in children younger than 5 years hospitalized for AGE from 2012 through 2017 in Hohhot, China.

## Methods

### Specimen collection

Between January 2012 and December 2017, 1863 fecal samples were collected from children younger than 5 years admitted to Maternal and Child Health Hospital in Hohhot with acute gastroenteritis (AGE). AGE was defined as two or more loose stools within a 24-h period. Stool specimens were collected within 7 days after the onset of the disease. All AGE cases admitted to the hospital were enrolled. All specimens were sent to the Inner Mongolia Centers for Disease Control and Prevention for NV detection, and the NV-positive samples were sent to the Institute for Viral Disease Control and Prevention, China CDC, for further testing and sequencing. Demographic and clinical data were collected using a standard questionnaire. Oral informed consent was obtained from the guardians of all hospitalized children for samples and data collection.

### RNA extraction and viral detection

Stool samples were prepared with phosphate-buffered saline solution to a final concentration of 10%. Viral RNA was extracted from 200 μL of a 10% stool suspension using the Viral Nucleic Acid Extraction Kit II (Geneaid, China), according to the manufacturer’s instructions. The RNA was then subjected to one-step real-time RT-PCR targeting NV sequences at the ORF1/ORF2 junction for NV detection, as described previously [25].

### Capsid and RdRp gene amplification

Following NV detection, positive samples were tested by conventional RT-PCR for amplification of the partial RdRp and VP1 gene regions of NV using the QIAGEN One-Step RT PCR Kit (QIAGEN). The 5′-end capsid gene (region C in ORF2) was amplified with oligonucleotide primer sets G1SKF/R and COG2F/G2SKR for 330 bp of the GI and 344 bp of the GII capsid genes respectively, as previously described [26]. The 3′-end of the RdRp gene (region A in ORF1) was amplified using a published oligonucleotide primer set, P290/P289 for a 319-bp fragment, as previously described [27]. The PCR products were visualized on agarose gels.

### Sequencing and phylogenetic analysis

All PCR products were purified and sequenced by Invitrogen Trading (Shanghai). The sequence identity was searched using the Basic Local Alignment Search Tool (BLAST) (https://blast.ncbi.nlm.nih.gov/Blast.cgi) multiple sequence alignment, and a phylogenetic tree was constructed using MEGA 7 [28]. The dendrogram was determined using the neighbor-joining method in MEGA7. Bootstrap resampling (1000 replications) was used and bootstrap values ≥75% are shown. The partial nucleotide sequences determined in this study were submitted to GenBank (acc. no. MK319217 to MK319535).

### Statistical analysis

Data were analyzed using SPSS 19.0 and Excel (MS office 2007). Numbers and percentages were computed for categorical variables. Categorical variables and proportions were compared using a two-sided chi-square test or Fisher’s exact test where appropriate. The 95% confidence intervals (95% CI) were estimated using the binomial exact method and *P*-values < 0.05 were considered statistically significant.

## Results

### Epidemiological characteristics of NV in AGE in Hohhot

From January 2012 to December 2017, 1863 AGE cases were enrolled in our study (402, 370, 223, 322, 251, and 295 in 2012 to 2017, respectively). Males accounted for 60.98% (1136/1863) and females 39.02% (727/1863). The median age was 8.0 (range 0–59) months (Table 1).

**Table 1:** Demographic characteristics of acute gastroenteritis patients admitted to the sentinel hospital in Hohhot, in 2012–2017

Characteristics	Total Tested	No. of norovirus Positive	Rate of Norovirus positive	*P* value
Participants	1863	450	24.15%	
Gender, *n* = 1863				
Males	1136	287	25.26%	*P* = 0.162
Females	727	163	22.42%	
Age group (month), *n* = 1863				
0–11	1258	308	24.48%	*P* = 0.072
12–23	483	106	26.67%	
24–35	75	20	22.95%	
36–47	32	8	25.00%	
48–59	15	8	53.33%	
Year, *n* = 1863				
2012	402	88	21.89%	*P* = 0.001
2013	370	87	23.51%	
2014	223	40	17.94%	
2015	322	106	32.92%	
2016	251	55	21.91%	
2017	295	74	25.08%	

**Fig. 1:**
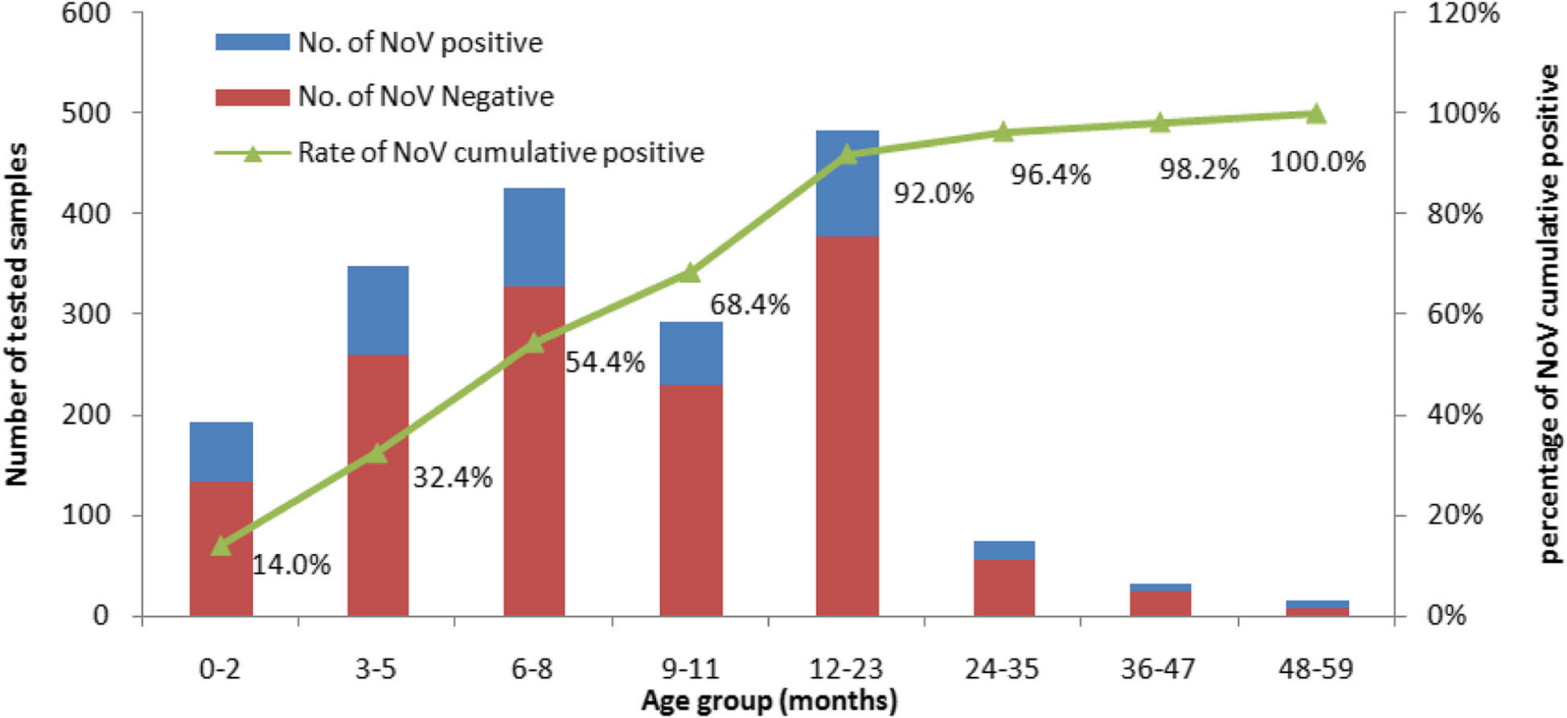
Age distribution of NV cases in children aged < 5 years in Hohhot, 2012–2017. The tested sample number (left *y-*axis) and the percentage (right *y-*axis) of NV cumulative positive cases isolated from stool samples and collected from children presenting with acute gastroenteritis in Hohhot are plotted against age groups (*x*-axis)

Overall, the NV positivity rate was 24.15% (450/1863). From 2012 to 2017, NV positivity ranged from 17.94 to 32.92%. The highest positivity was observed in 2015 (Table 1). Gender was not significantly associated with NV infection (male/female, *χ*^*2*^ = 1.956, *P* = 0.162). The detection rates for children 0–11, 12–23, 24–35, 36–47, and 48–59 months were 24.48% (308/1258), 26.67% (106/483), 22.95% (20/75), 25% (8/32), and 53.33% (8/15), respectively, and they were not significantly different (*χ*^*2*^ = 8.602, *P* = 0.072). However, the total number of AGE cases and number of NV cases in the 0- to 23-month age group were much higher than those of the 24- to 47-month age group. Of NV AGE, 14% occurred before the age of 3 months. By the age of 24 months, nearly 97% of NV-positive cases had occurred. The cumulative NV-positive rate by age indicated that 0–23 months was the main age group for NV infection (Fig. 1).

**Fig. 2:**
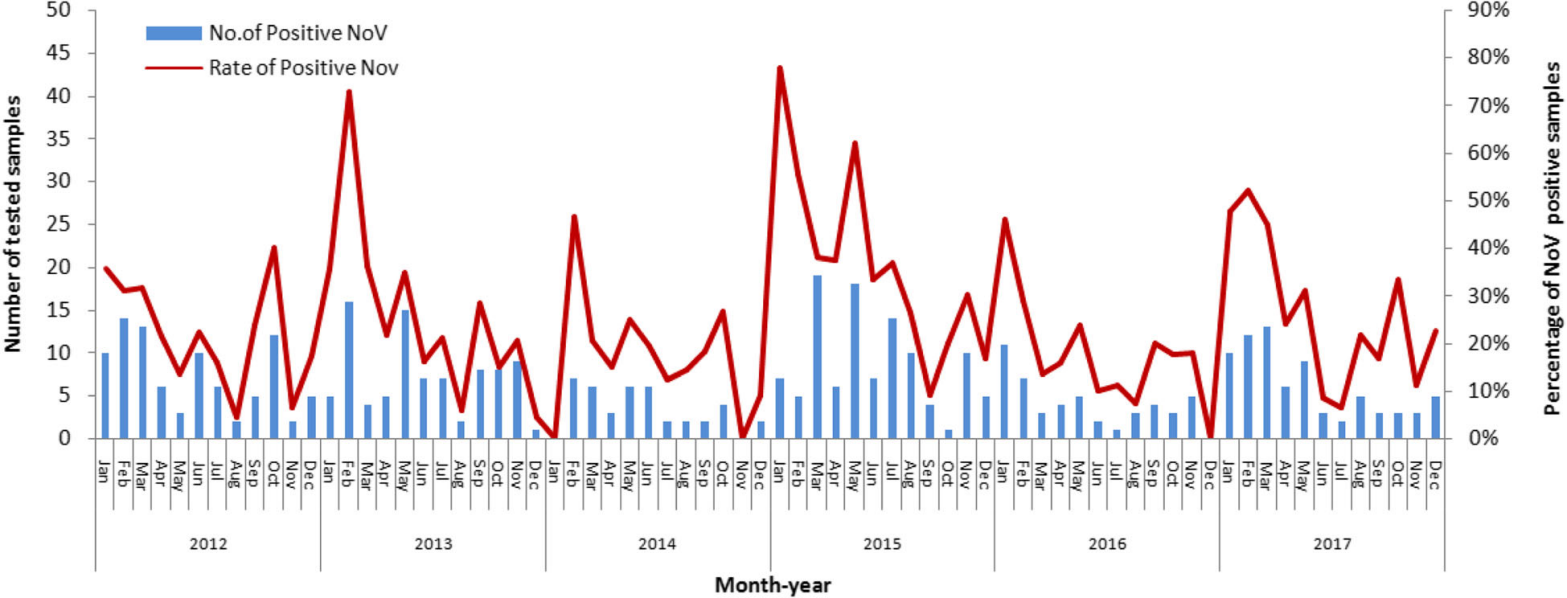
Seasonal distribution of NV cases among children aged < 5 years in Hohhot. The absolute number (left *y-*axis) and percentage (right *y-*axis) of NV-positive cases isolated from stool samples and collected from children presenting with acute gastroenteritis in Hohhot are plotted against the month and year (*x*-axis)

NV infection can occur throughout the year (Fig. 2). Every year, NV infection had three peaks. The highest peak was from January to March, with 40~80% positivity. Two small peaks occurred in May and September, with 30% positivity.

### Genotyping of NV

Of the 450 NV-positive samples, 332 (74%) were genotyped. NV geno-grouping revealed that all belonged to GII and none belonged to GI. Of the 332 genotyped samples, both the RdRp and capsid genes were genotyped in 296 samples, only the RdRp gene was determined in 26 samples, and only the capsid gene in 10 samples. Based on the capsid gene, strain GII.3 was predominant with 71.24% (218/306) prevalence, followed by strains GII.4 (23.53%, 72/306), GII.2 (2.61%, 8/306), GII.13 (1.63%, 5/306), GII.6 (0.65, 2/306), and GII.5 (0.33%, 1/306). Based on the RdRp gene, GII.P12 was most prevalent at 71.42% (230/322), followed by GII. Pe (12.42%, 32/322), GII.P4 (11.18%, 36/322), GII.P16 (4.04%, 13/322), GII.P7 (0.62%, 2/322), and GII.P22 (0.31%, 1/322).

The analysis of the 296 sequences of both the RdRp and capsid genes showed that at least 11 RdRp/capsid NV genotypes circulated in Hohhot during the last 6 years (Table 2). The most common genotype was the GII.12/GII.3 recombinant (69.6%), followed by the GII.Pe/GII.4 (13.2%) and GII.P4/GII.4 (9.8%) recombinants. The remaining identified recombinant genotypes (GII.P16/GII.2, GII.P12/GII.4, GII.P16/GII.4, GII.P4/GII.3, GII.P16/GII.13, GII.P12/GII.13, GII.P12/GII.12, and GII.P22/GII.5) were uncommon.

**Table 2:** Distribution of norovirus RdRp and capsid genotypes among children aged < 5 years in Hohhot, 2012–2017

Genotype			Total recombinants	Proportion of recombinants
Capsid		RdRp		
GII.4	the New Orleans 2010 variant	GII.P4	2	9.8%
	2006b variant		13	
	Sydney 2012 variant		14	
	2006b variant	GII.Pe	1	13.2%
	Sydney 2012 variant		38	
	Sydney 2012 variant	GII.P12	2	0.7%
	Sydney 2012 variant	GII.P16	1	0.3%
GII.3		GII.P12	206	69.6%
		GII.P4	3	1.0%
GII.2		GII.P16	8	2.7%
GII.13		GII.P16	4	1.4%
		GII.P12	1	0.3%
GII.5		GII.P22	1	0.3%
GII.6		GII.P7	2	0.7%
Total			296	

**Fig. 3:**
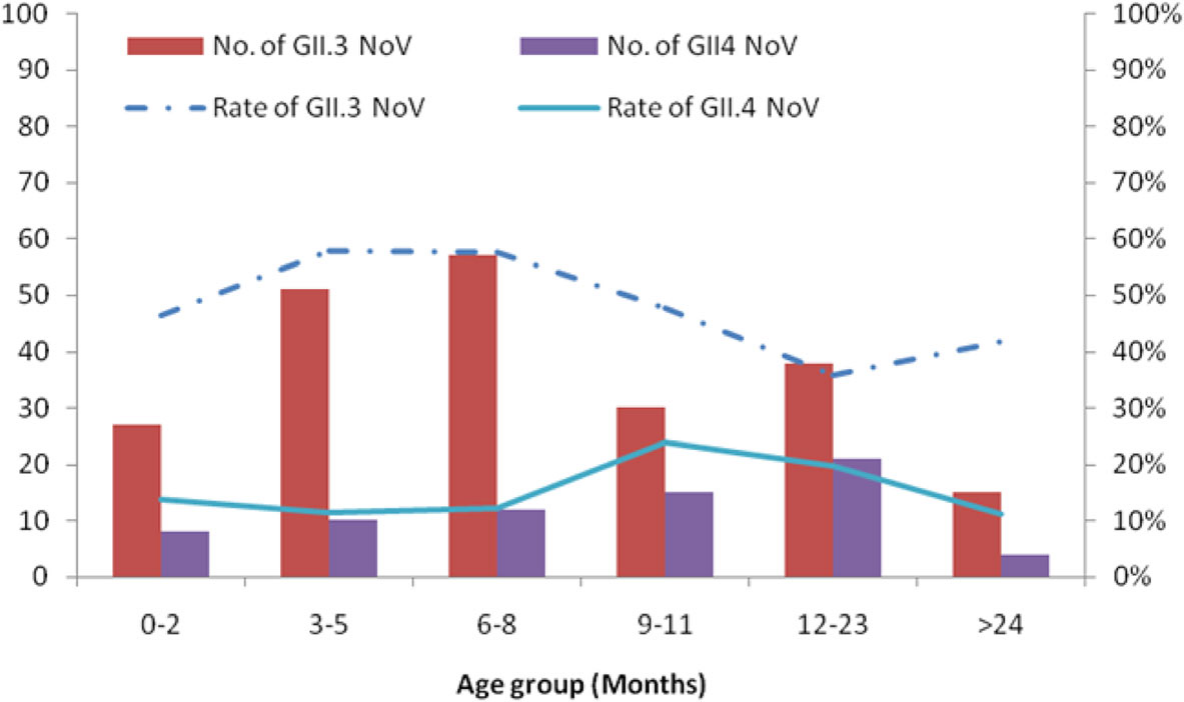
Age group distribution (months) of children aged < 5 years hospitalized for AGE caused by NV GII.3 and GII4 in Hohhot, 2012–2017. The percentages were calculated with respect to total NV of each analyzed genotype

Ninety-two percent (414/450) of the NV cases occurred in the 0- to 23-month age group. Further analysis showed a difference between the age distributions of GII.3- and GII.4-positive children (Fig. 3). From 2012 to 2017, 78% of the GII.3 infections occurred in children aged 0–11 months, while 72% of the GII.4 infections occurred in children aged 6–24 months. Children infected with GII.3 were younger (median 7.0 months) than GII.4-positive patients (median 10.0 months) (*P* = 0.021, Mann-Whitney U test).

### Phylogenetic analysis of NV

**Fig. 4:**
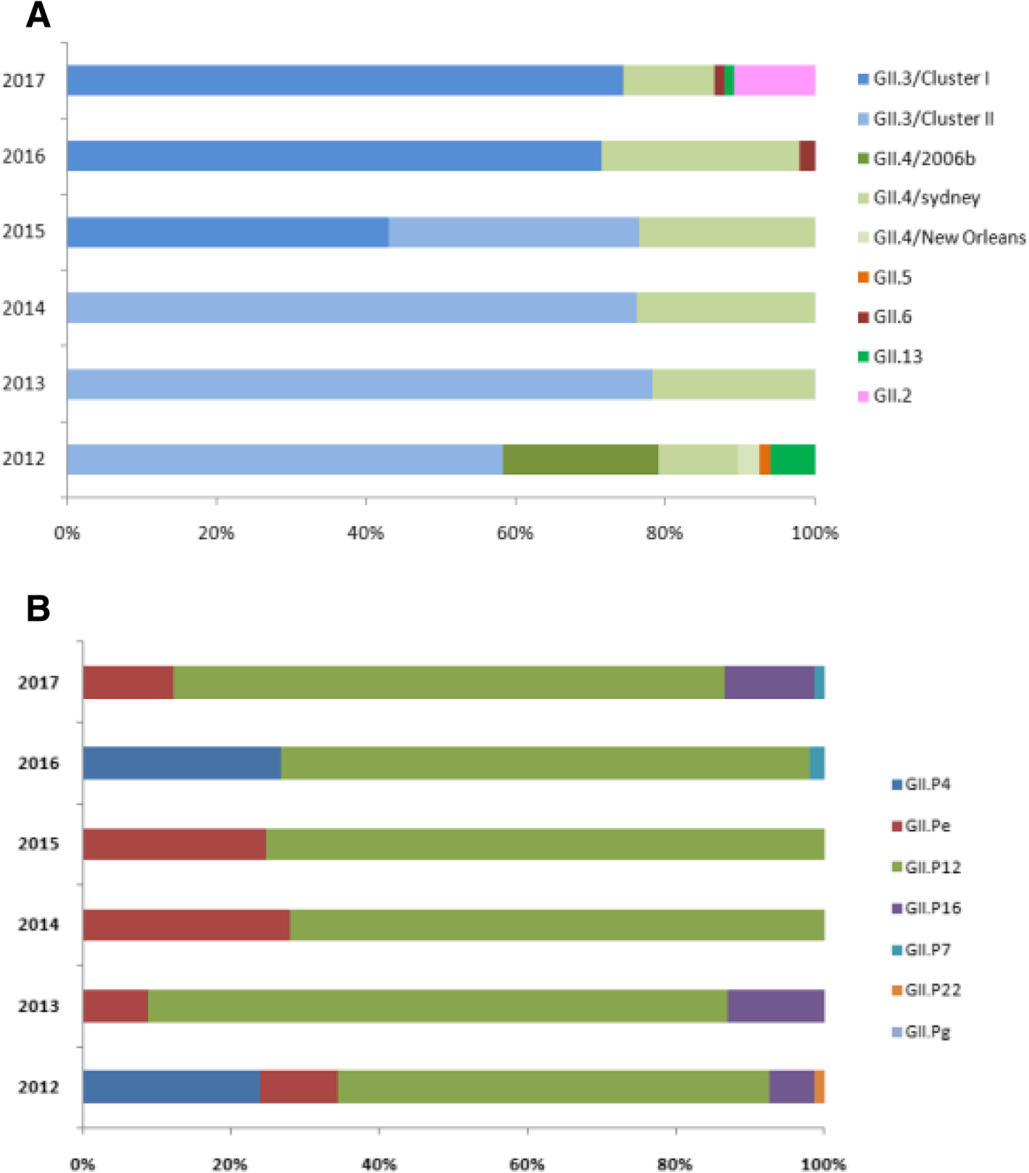
Distribution of NV capsid genotypes (A) and RdRp genotypes (B) by year among children aged < 5 years in Hohhot, 2012–2017

**Fig. 5:**
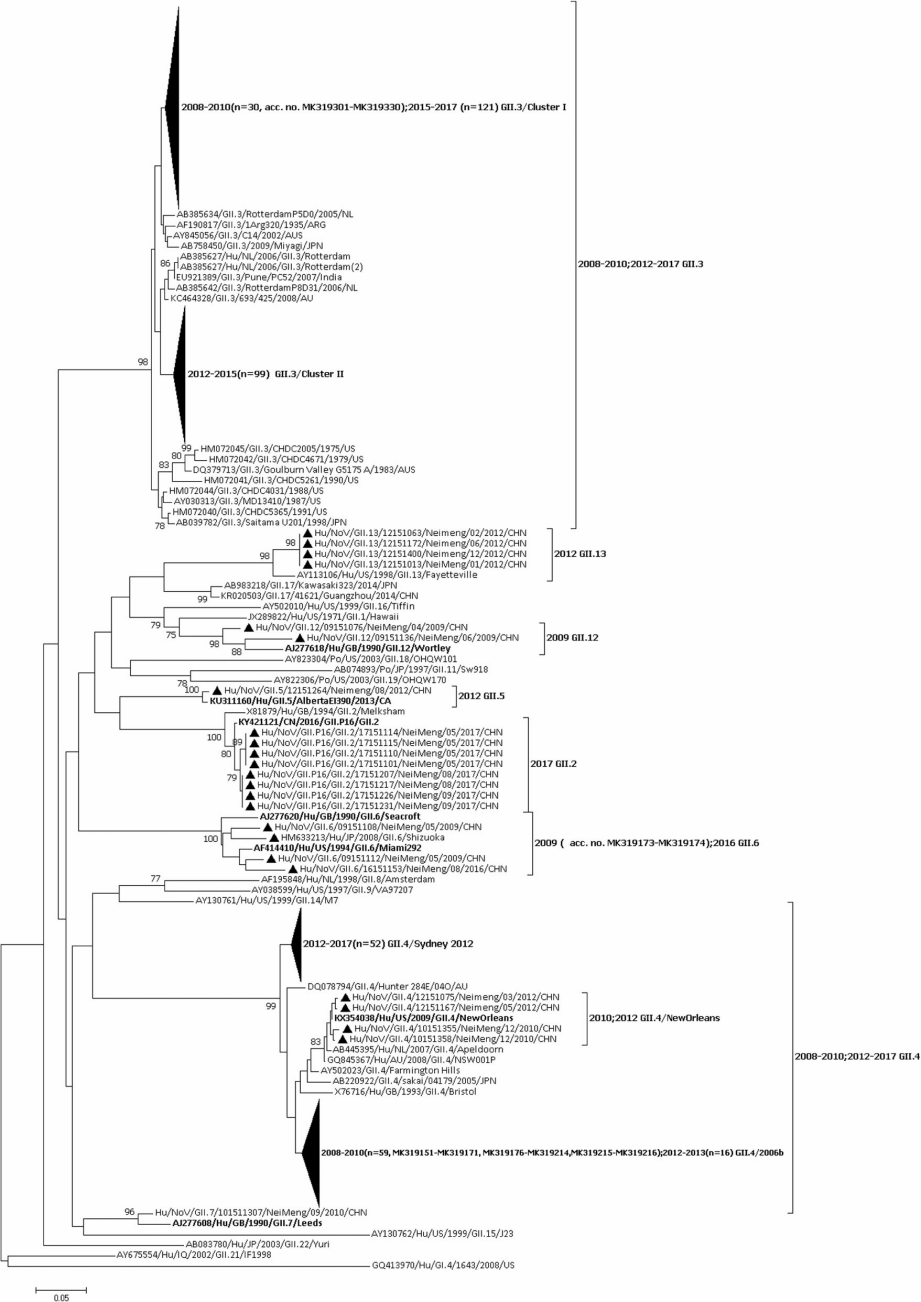
Phylogenetic analysis of the NV viral protein 1 major capsid gene. Nucleotide sequences spanning nucleotides 5081–5362 (length = 282 nt) of NVs isolated in Hohhot were aligned with reference strains obtained from GenBank. The reference strains of Hohhot NoV strains from 2008 to 2010 are GenBank acc. Nos. MK319151–MK319216 and MK319301–MK319330. The phylogenetic tree was constructed based on the nucleotide sequences using the neighbor-joining method with bootstrap analysis of 1000 replicates using MEGA ver. 7.0. Bootstrap values > 75% are shown

In Hohhot, GII.3 was the predominant genotype from 2012 to 2017, accounting for 58, 78, 76, 75, 71, and 74% in the respective years (Fig. 4). Phylogenetic analysis of the partial capsid gene sequences demonstrated that all of the Hohhot GII.3 strains (*n* = 248) grouped into two clusters (I and II) (Fig. 5). We obtained 30 reference sequences of Hohhot NoV GII3 strains from 2008 to 2010 in GenBank (acc. no. MK319301–MK319330), which belong to GII.3/cluster I. Whereas the GII.3/cluster II strain was the main epidemic strain in 2012–2014, GII.3/cluster I was not detected. Nevertheless, in 2015, the GII.3/cluster I strain began to re-emerge and became common simultaneously with the GII.3/cluster II strain. From 2016 to 2017, the former became the main epidemic strain and the latter was not detected. Based on the RdRp genotype (Table 2), the GII.3/cluster I and GII.3/cluster II strains were both detected in combination with GII.P12 and GII.P4, and the latter was detected in combination with GII. Pe and GII.P6.

In Hohhot, GII.4 was the second-most dominant genotype from 2012 to 2017, accounting for 34, 22, 24, 23, 27, and 12% in the respective years (Fig. 4). During the study period, three variants of the GII.4 genotype were identified. Fourteen identified GII.4 strains were clustered closely with the GII.4/2006b variant, sharing 98.1–100% identity with the AB291542/GII.4/Kobe034/2006/JAN strain. Fifty-six identified with the GII.4/Sydney 2012 variants and shared 98.1–100% identity with the JX459908/GII.4/Sydney/2012/AU strain. Two were the GII.4/New Orleans 2009 variant, sharing 99.3 and 99.7% with the KX354038/GII.4/New Oreans/2009/US strain, respectively. We obtained 61 reference sequences of Hohhot NV GII.4 strains from 2008 to 2010 in GenBank (acc. no. MK319151–MK319171, MK319176–MK319214, and MK319215–MK319216), 59 of which belonged to the GII.4/2006b variant, and 2 to the GII.4/New Orleans 2009 variant. In 2012, the predominant strain was the GII.4/2006b variant, but after 2013, the GII.4/Sydney 2012 variant replaced the GII.4/2006b variant as the epidemic strain, and the GII.4/2006b variant was not detected. Based on the RdRp genotype (Table 2), the GII.4/2006b variant was detected in combination with GII.P4; the GII.4/Sydney 2012 variant was detected in combination with GII. Pe, GII.P4, GII.P12, and GII.P6; and the GII.4/New Orleans 2009 variant was detected in combination with GII.P4.

## Discussion

In the present study, we conducted long-term surveillance for NV-associated gastroenteritis in hospitalized children younger than 5 years of age in Hohhot, Inner Mongolia, China. From 2012 to 2017, the NV positivity rate was 24.15% in Hohhot, which was close to that in Wuhan (2007–2010, 25.9%), but higher than that in other parts of China [29]. This result indicates the high activity of NV in recent years in Hohhot. Furthermore, 93% (1741/1863) of AGE cases were children aged 0 to 23 months, and NV positivity was higher in the 0–23-month age group than in children older than 24 months, which implies that young children are more vulnerable to NV infection. This result is consistent with previous studies [15, 22, 30, 31], indicating that intervention approaches such as a vaccine for NV infection should be implemented for children under 24 months of age.

According to a systematic review of the global seasonality of NV, in the Northern Hemisphere NV usually peaks in cold seasons, whereas it does not exhibit a consistent summer or winter peak in the Southern Hemisphere [32]. In southeastern China, NV infections were detected mainly in the spring and summer [26], whereas in North China, NV infections occurred commonly in the winter [33, 34]. However, in this study we found three peaks of NV infection per year in Hohhot. The highest peak was observed in winter, and two small peaks occurred in spring and fall. Hohhot is located in the northern part of China in the continental Mongolian plateau, with short, hot, rainless summers, and long, severe, cold, snowless winters. This differs from the climate in the other northern regions in China. The seasonal pattern of NV in Hohhot might be related to this unique local climate.

The oldest GII.3 NV strain identified to date is from a 1983 sample isolated in the Goulburn Valley of Australia [13], and GII.3 has been reported to be the major cause of sporadic pediatric NV infection [15, 17, 35, 36]. In China, early reports on NV showed GII.3 was prevalent in 1999 [37, 38], and until now, GII.3 was reported as the dominant genotype next to GII.4 in sporadic NV infection [39, 40]. However, in this study, we found that NV GII.3 was the most prevalent strain among children hospitalized for AGE from 2012 to 2017. Hohhot, located in the North China Plateau, is vast and sparsely populated, and lags behind the developed cities of our country in socioeconomic development and the convenience of transportation. Population flow and economic trade in Hohhot are infrequent. During this period, the GII.P12/GII.3 recombinant was the most common genotype, and it was also detected more frequently in China and Japan than in other parts of the world [14, 34, 36]. The GII.3 strains identified in our study were grouped into two clusters (I and II) in a phylogenetic analysis of the capsid gene sequences. Lacking early NV surveillance data from Hohhot, it is not yet possible to determine whether GII.3/cluster II strains emerged and spread before 2012. However, our results suggested the hypothesis of an alternating epidemic pattern between GII.3/cluster I strains and GII.3/cluster II strains in Hohhot. Because only partial capsid gene sequences were obtained, rather than the complete VP1 region, or even the longer genome including the ORF1/ORF2 overlap region, we cannot clearly delineate the evolutionary characterization of GII.3 strains; this gap in knowledge should be addressed in the future.

Furthermore, a broad genetic diversity of NV GII 4 genotypes among children hospitalized for AGE was found in Hohhot. From 2006 to 2011, the GII.4/2006b variant was the predominant cause of sporadic NV cases and outbreaks in China and Japan [41–43]. However, this strain was replaced by the GII.4/Sydney 2012 strain in October 2012, and this strain caused a worldwide increase in NV outbreaks [44]. GII.4/Sydney 2012 was first identified in Australia, France, and New Zealand, and subsequently isolated in the United States, Belgium, Denmark, Scotland, Japan, and China. Of note, after 2012, the GII.4/Sydney 2012 variant was the dominant epidemic strain in Hohhot and most other regions of China, both in sporadic NV cases and NV outbreaks [19, 21]. Previous studies show GII.4 usually caused epidemics in adult populations, whereas GII.3 NV was prevalent in young children and infants [45, 46]. The reasons for the difference between GII.4 and GII.3 are still unknown. Perhaps a variety of factors account for it, including cross protection from heterologous strains or the minimal infectious dose necessary to cause disease. More studies are needed to address this issue.

Unlike neighboring cities and other parts of China, where emerging GII.P17-GII.17 NVs caused outbreaks during 2014–2015, no GII.P17-GII.17 was found in children hospitalized for AGE in Hohhot in 2015. Previous reports showed that infection with the emergent GII.P17-GII.17 was more likely to occur in older children and adults [47], which may be the reason for negative detection of GII.P17-GII.17 in Hohhot, with 93% of AGE cases among children ages 0 to 23 months. However, the exact mechanism for the emergence, prevalence, and age preference of GII.P17-GII.17 remains unknown.

NV recombination was genetically characterized via analysis of regions from both the capsid gene and RdRp gene. In our study, lacking the overlap genes from the RdRp and VP1 regions, the NV recombinants were not conformed and may actually have come from two different coinfecting NV genotypes. However, since 2018, a conventional RT-PCR, as developed by the US Centers for Disease Control and Prevention (Atlanta, GA, USA) (J. Vinjé, pers. comm., 2017 Mar 1, [48], has been used for amplification of partial RdRp and VP1 gene regions. This RT-PCR will be used in our future NV surveillance.

## Conclusions

We found high NV prevalence in children younger than 5 years in Hohhot who were hospitalized for AGE. GII.3 NV was identified as the most common cause of AGE from 2012 to 2017, and circulating genotypes changed over time with the emergence of new genetic variants. Continuous monitoring with molecular genotyping is necessary for NV diseases in children and will be helpful for the local control and prevention of AGE.

## Data Availability

The datasets used and/or analyzed during the current study are available from the corresponding authors on reasonable request.

## Abbreviations

AGE: acute gastroenteritis
NV: norovirus
ORFs: open reading frames
RdRp: RNA-dependent RNA polymerase
real-time RT-PCR: real-time reverse transcription polymerase chain reaction
